# Metabolomics in paraganglioma: applications and perspectives from genetics to therapy

**DOI:** 10.1530/ERC-22-0376

**Published:** 2023-05-11

**Authors:** Susan Richter, Timothy J Garrett, Nicole Bechmann, Roderick J Clifton-Bligh, Hans K Ghayee

**Affiliations:** 1 Institute for Clinical Chemistry and Laboratory Medicine, University Hospital Carl Gustav Carus, Medical Faculty Carl Gustav Carus, Technische Universität Dresden, Fetscherstrasse, Dresden, Germany; 2 Department of Pathology, Immunology, and Laboratory Medicine, College of Medicine, University of Florida, Gainesville, Florida, USA; 3 Cancer Genetics Laboratory, Kolling Institute, Faculty of Medicine and Health, The University of Sydney, St Leonards, Australia; 4 Department of Endocrinology, Royal North Shore Hospital, St Leonards, Australia; 5 Department of Internal Medicine, Division of Endocrinology, University of Florida College of Medicine and Malcom Randall VA Medical Center, Gainesville, Florida, USA

**Keywords:** mass spectrometry, nuclear magnetic resonance spectroscopy, succinate dehydrogenase, fumarate hydratase, oncometabolites

## Abstract

Metabolites represent the highest layer of biological information. Their diverse chemical nature enables networks of chemical reactions that are critical for maintaining life by providing energy and building blocks. Quantification by targeted and untargeted analytical methods using either mass spectrometry or nuclear magnetic resonance spectroscopy has been applied to pheochromocytoma/paraganglioma (PPGL) with the long-term goal to improve diagnosis and therapy. PPGLs have unique features that provide useful biomarkers and clues for targeted treatments. First, high production rates of catecholamines and metanephrines allow for specific and sensitive detection of the disease in plasma or urine. Secondly, PPGLs are associated with heritable pathogenic variants (PVs) in around 40% of cases, many of which occur in genes encoding enzymes, such as succinate dehydrogenase (SDH) and fumarate hydratase (FH). These genetic aberrations lead to the overproduction of oncometabolites succinate or fumarate, respectively, and are detectable in tumors and blood. Such metabolic dysregulation can be exploited diagnostically, with the aim to ensure appropriate interpretation of gene variants, especially those with unknown significance, and facilitate early tumor detection through regular patient follow-up. Furthermore, *SDHx* and *FH* PV alter cellular pathways, including DNA hypermethylation, hypoxia signaling, redox homeostasis, DNA repair, calcium signaling, kinase cascades, and central carbon metabolism. Pharmacological interventions targeted toward such features have the potential to uncover treatments against metastatic PPGL, around 50% of which are associated with germline PV in *SDHx*. With the availability of omics technologies for all layers of biological information, personalized diagnostics and treatment is in close reach.

## Introduction

The metabolome constitutes the entirety of metabolites present in a cell and is the result of the cell’s genetic, transcriptomic, and proteomic characteristics. Thereby, metabolism is responsible for maintaining life by conversion of energy in a form suitable for cellular reactions, generation of cellular building blocks, and removal of end products. Nevertheless, metabolites are not only important as precursors and products of enzymatic reactions, but they can also act as signaling molecules in the cell as well as extracellularly as paracrine or/and endocrine factors. Examples are succinate and lactate; both act through G protein-coupled receptors and regulate inflammatory responses (Kes *et al.* 2020). The amino acids glycine, glutamate, and γ-aminobutyric acid are also players in central carbon metabolism and additionally function as neurotransmitters. Intracellularly, metabolites regulate enzymatic reactions, influence gene expression through the modulation of epigenetic processes, for example through the so-called oncometabolites 2-hydroxyglutarate, succinate, and fumarate, and alter protein function and stability through posttranslational modifications, one well-known example being the regulation of hypoxia-inducible factor (HIF) proteins (Liu & Wellen 2020).

**Figure 1:**
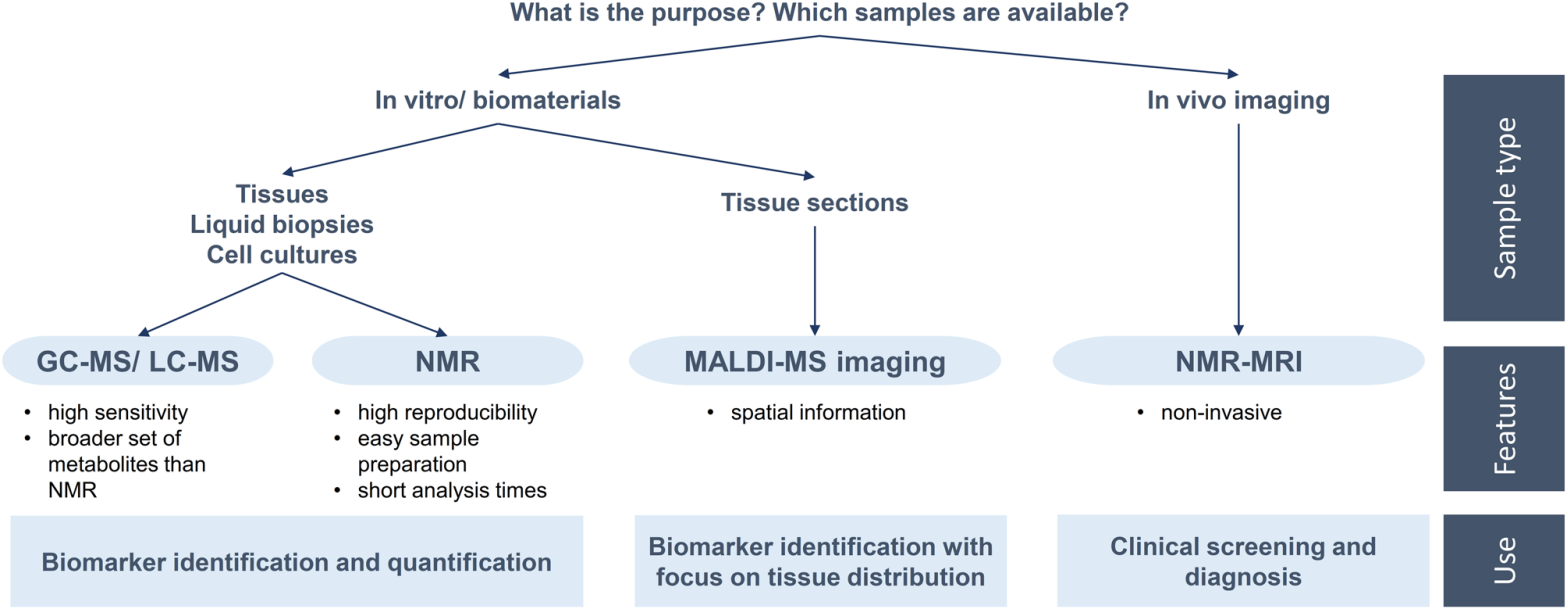
How to choose your metabolomics method. GC-MS, gas chromatography with mass spectrum; LC-MS, liquid chromatography with mass spectrometry; MALDI-MS, matrix-assisted laser desorption/ionization mass spectrometry; MRI, magnetic resonance imaging; NMR, nuclear resonance spectroscopy.

Metabolites comprise diverse groups of chemical classes, from small organic acids to derivatives of amino acids, complex ring structures, and long carboxyl chains. The Human Metabolome Database (HMDB) lists more than 220,000 endogenous metabolites. These diverse chemical structures dictate the analytical approach necessary for detection. Broadly speaking, there are two techniques used for the quantification of metabolites, nuclear magnetic resonance (NMR) spectroscopy and mass spectrometry (MS) coupled either to gas chromatography or to liquid chromatography (LC). Targeted approaches focus on a defined group of metabolites, while untargeted analyses detect a wider spectrum of metabolites. Depending on the goal of the analysis, the appropriate technique has to be selected (Fig. 1). NMR relies on inherent magnetic properties of atomic nuclei to identify compounds and their structures, whereas MS detects the mass-charge-ratio (*m*/*z*) and identifies compounds based on their characteristic fragmentation in a targeted approach or by their mass accuracy in an untargeted approach (Markley *et al.* 2017). MS usually provides better sensitivity as well as a much broader set of metabolites and lipids that can be measured compared to NMR. NMR has the advantage of easy sample preparation without any requirement for chemical derivatization or downstream chromatographical separation as well as short measuring times and very high reproducibility. Furthermore, both methodologies can be linked to imaging modalities. NMR combined with anatomical imaging by magnetic resonance imaging (MRI) is a tool to non-invasively measure metabolites in patient tissues *in vivo*. Matrix-assisted laser desorption ionization (MALDI)-MS imaging can be applied to tissue sections to visualize the spatial distribution of metabolites.

For metabolomics using MS, the approaches (targeted or untargeted) are typically conducted on different instruments. For targeted metabolomics, most methods rely on the use of triple quadrupole instrumentation and are operated under selected reaction monitoring (SRM) or multiple reaction monitoring (MRM), a form of tandem MS/MS. In these modes, the precursor metabolite is isolated in the first quadrupole and then subjected to collision-induced dissociation in the collision cell, which induces fragmentation causing product ions to be formed. In the next quadrupole, one or more product ions are isolated. Thus, the reported measurement is a precursor/product ion pair that is associated with each metabolite, allowing for a very specific and confident identification of each metabolite. An example is the analysis of catecholamine metabolites in the diagnosis of pheochromocytoma/paraganglioma (PPGL) (Eisenhofer & Peitzsch 2014). A triple quadrupole operating in the SRM/MRM mode is typically the most sensitive approach for measurements and is often employed when very low levels of an analyte are expected. Such a targeted method is limited to only the metabolites for which SRM/MRM pairs are assigned and thus cannot expand to new metabolites that could be biomarkers of disease or expand on our knowledge of metabolism.

Untargeted or global metabolomics most often relies on high-resolution mass spectrometry using a quadrupole-time-of-flight or an Orbitrap mass spectrometer that can measure masses with high accuracy, typically 5 ppm or less (Xian *et al.* 2012). Metabolites are identified either by searching databases such as the HMDB using the mass accuracy of the instrument or by developing an internal library that combines both *m*/*z* and retention time from chromatographic separation (Alseekh *et al.* 2021). In this manner, a metabolite is represented by the *m*/*z* and retention time pair, which is often called a feature. Statistical analysis relies on comparing the same signal across multiple samples in the peak list to identify metabolites that are significantly different between the groups. Not all metabolites can be identified, and some features remain ambiguous, representing one of the main challenges in metabolomics. Nevertheless, metabolomics has the potential to identify new metabolic pathways, shed light on disease states, or identify new biomarkers for diagnosis.

For targeted and untargeted LC-MS approaches, the choice of chromatography is essential to achieve specificity and sensitivity. The two most common separation approaches for LC are reversed-phase (RP) and hydrophilic interaction liquid chromatograph (HILIC). RP is the most common approach in metabolomics as it provides the best peak capacity and reliability covering semi-polar to non-polar metabolites. The performance on polar analytes is limited, even for polar embedded stationary phases. HILIC is a nowadays more widely used alternative to RP, because it can be employed to separate very polar analytes, like sugars or sugar-containing metabolites. Both LC methods are highly compatible with electrospray ionization (ESI) as they use a combination of water, methanol, and/or acetonitrile as mobile phases. Alternatively, atmospheric pressure chemical ionization (APCI) can be used. ESI is better suited for more polar, non-volatile molecules, while APCI has an advantage for non-polar, volatile compounds. MALDI as a third ionization approach most often is applied in imaging MS.

## PPGL and catecholamine metabolism

Non-epithelial neuroendocrine neoplasms originating from neural crest-derived cells are referred to as paragangliomas according to the fifth series of the WHO Classification of Endocrine and Neuroendocrine Tumours (Mete *et al.* 2022). They can arise either along sympathetic and parasympathetic ganglia (PGL) or in the adrenal medulla (pheochromocytomas, PHEO). These rare and mostly catecholamine-producing tumors can lead to diverse symptoms related to either mass effects or the actions of catecholamines on blood pressure, heart rate, sweating and other body functions (Prejbisz *et al.* 2011, Zuber *et al.* 2011). Life-threatening complications, such as hypertensive crisis, can occur when PPGLs are undiagnosed during surgical procedures (Riester *et al.* 2015).

The catecholamine metabolism with high production rates in PHEO and sympathetic PGL tumor cells can be exploited diagnostically and aid in patient stratification according to underlying genetic drivers. As this topic was reviewed extensively in previous publications, we direct the reader to the following reviews (Eisenhofer *et al.* 2004, Eisenhofer *et al.* 2017). In chromaffin cells of the adrenal, catecholamines are not continuously secreted; instead, they are stored in vesicles waiting for stimuli that trigger secretion. In many PPGLs, catecholamines are released in intervals leading to fluctuating plasma levels. Importantly though, degradation of about 10% of catecholamines is a continuous process that takes place within chromaffin cells. Degradation products, the so-called metanephrines, hence reflect catecholamine production rates of PPGLs. Targeted LC-MS/MS-based measurements of metanephrines in either plasma or urine show high diagnostic sensitivity and specificity and are considered gold standard in the diagnostic workup to exclude or confirm PPGLs (Eisenhofer & Peitzsch 2014, Lenders *et al.* 2014).

Further research in this area identified the dopamine metabolite 3-methoxytyramine as a plasma biomarker for metastatic PPGL (Eisenhofer *et al.* 2012). Higher levels of 3-methoxytyramine are indicative of shorter disease-specific survival in patients with parasympathetic PGL, also called head-and-neck PGL, or metastases (Pamporaki *et al.* 2022). Head-and-neck PGLs produce only very low levels of catecholamines; hence, metanephrines are for the most part within normal limits for these patients (Richter *et al.* 2022). With improved assay sensitivity, however, plasma 3-methoxytyramine might be useful for screening of head-and-neck PGLs in the future. *In situ* derivatization, for example, enabled the simultaneous measurement of several amines with increased assay sensitivity for 3-methoxytyramine by LC-MS/MS (van Faassen *et al.* 2020). The use of such extended metabolite panels might identify further markers for patient stratification.

## PPGL genetics and tumorigenic effects of oncometabolites

PPGLs have the highest number of hereditary cases amongst all neoplasms. About 40% arise due to germline pathogenic variants (PVs) in 1 of 21 known susceptibility genes. A majority of these hereditary PPGLs are associated with PVs in one of five genes, each connected to a specific syndrome: the von Hippel–Lindau (*VHL*) gene, rearranged during transfection (*RET*) protooncogene, the neurofibromatosis type 1 (*NF1*) gene, and succinate dehydrogenase (SDH) genes *SDHB* and *SDHD*. Less commonly, PVs occur in other SDH genes: *SDHA*, *SDHC*, and assembly factor *SDHAF2*, as well as in transmembrane protein 127 (*TMEM127*), MYC associated factor X (*MAX*), fumarate hydratase (*FH*), mitochondrial malate dehydrogenase (*MDH2*), succinate-CoA ligase GDP-forming subunit beta (*SUCLG2*), solute carrier family 25 (*SLC25A11*), mitochondrial glutamic-oxaloacetic transaminase (*GOT2*), dihydrolipoamide S-succinyltransferase (*DLST*), Egl nine homolog 1 and 2 (*EGLN1/2*), kinesin family member 1B (*KIF1B*), DNA methyltransferase 3α (*DNMT3A*), and endothelial PAS domain-containing protein 1 (*EPAS1* or *HIF2A*) (Jhawar *et al.* 2022). Additionally, somatic mutations in these or other cancer genes were described, explaining the cause of roughly 80% of PPGLs (Curras-Freixes *et al.* 2017, Fishbein *et al.* 2017).

Since *SDHB* mutations are linked to a higher rate of metastatic disease and curative treatments are lacking, early tumor detection and removal are paramount to the prevention of metastases (Gimenez-Roqueplo *et al.* 2003, Davidoff *et al.* 2022). This has been recognized in clinical guidelines, in that patients who already have a tumor should undergo genetic testing to identify the underlying cause, and patients who have a confirmed mutation in an *SDHx* gene but no PPGL yet should undergo regular follow-up (Lenders *et al.* 2014, Amar *et al.* 2021). Besides PPGL, *SDHx* PVs predispose to renal cell carcinomas (Vanharanta *et al.* 2004) and gastrointestinal stromal tumors (Janeway *et al.* 2011). DNA sequencing technologies have been optimized to the point that the majority of gene variants can be detected by next-generation sequencing, either using a preselected panel of target genes or through sequencing the entire exome or genome. The problem, however, is that most gene variants are neutral and have no impact on health. Classifying gene variants according to their pathogenicity follows a standardized procedure that considers different information, including the nature of the mutation, *in silico* predictions, population frequency, and functional information (Richards *et al.* 2015). The latter becomes especially important for missense variants in disease-causing genes, since their effect on function is less predictable than for truncating variants. Metabolomics can contribute valuable information about gene variants of metabolic enzymes, such as *SDHx* and *FH*, by providing measurements of precursors and products and hence aids in the classification process.

**Figure 2:**
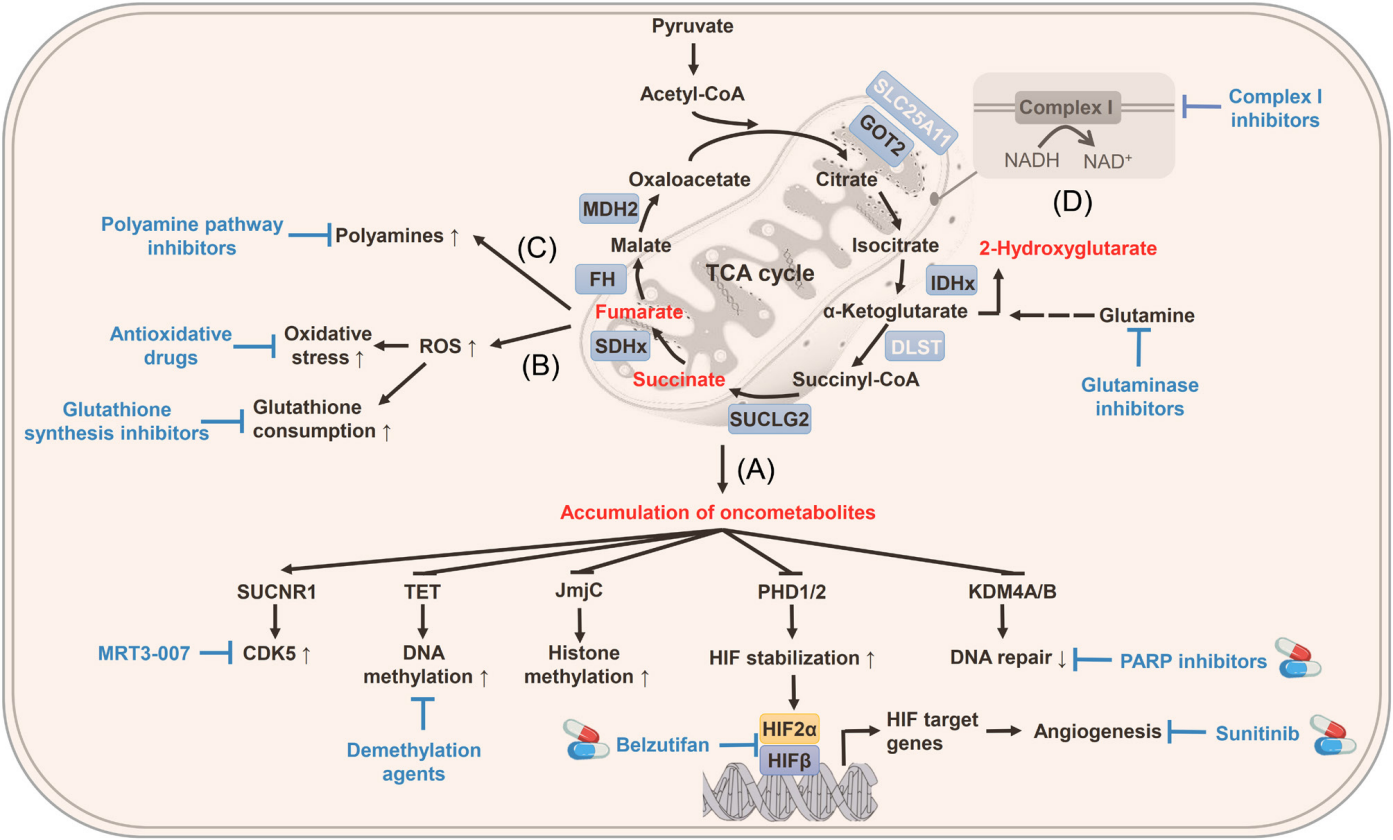
Cellular consequences of oncometabolites and targeted therapies for metastatic PPGL. (A) Oncometabolites, such as succinate, fumarate, and 2-hydroxyglutarate, change many cellular signaling pathways that can be potential treatment targets. Succinate stimulates the receptor SUCNR1 and results in the activation of cyclin-dependent kinase 5 (CDK5). Oncometabolites inhibit α-ketoglutarate-dependent enzymes, including DNA and histone demethylases (TET and JmjC, respectively), prolyl hydroxylases (PHD) that lead to hypoxia-inducible factor (HIF) stabilization, and lysine demethylases KDM4A/B that are involved in DNA repair. (B) Aberrations in mitochondrial metabolism increase the production of reactive oxygen species (ROS), resulting in increased oxidative stress and glutathione consumption as a mechanism of compensation. (C) Loss of SDH leads to increased polyamine synthesis and (D) dependence on glutamine usage as well as on respiratory complex I for oxidation of NADH to maintain flux through the Krebs cycle. Blue boxes depict PPGL-susceptibility genes involved in mitochondrial metabolism (other susceptibility genes are listed in the manuscript text): DLST, dihydrolipoyllysine-residue succinyltransferase; FH, fumarate hydratase; GOT2, glutamic-oxaloacetic transaminase; IDHx, isocitrate dehydrogenase 1 or 2; MDH2, malate dehydrogenase 2; SDHx, succinate dehydrogenase subunit genes; SLC25A11, mitochondrial α-ketoglutarate/malate carrier; SUCLG2, succinate-CoA ligase GDP-forming subunit β; gene mutations known to increase oncometabolites (red letters) are shown in black letters. Targeted therapies are highlighted in blue, with clinically approved therapies for other cancers marked with drug capsules.

Genetic alterations have amongst others also metabolic consequences in a cell (Fig. 2). Especially mutations affecting SDH, FH, or isocitrate dehydrogenase (IDH) lead to strong increases in succinate, fumarate, and 2-hydroxyglutarate, respectively. *IDH1* and *IDH2* hotspot mutations occur somatically in rare instances in PPGL (Richter *et al.* 2019, Li *et al.* 2022). Those oncometabolites cause global cellular changes by affecting epigenetic reprogramming and hypoxic signaling (Pollard *et al.* 2005, Yang & Pollard 2013). Similar to downstream consequences of *VHL* mutations, oncometabolites inhibit prolyl hydroxylases (PHDs), leading to accumulation of HIFs and subsequent activation of pathways associated with pseudohypoxia, which creates opportunities for angiogenesis and cellular proliferation under normal oxygen pressures. Additionally, histone lysine demethylases and enzymes of the ten-eleven translocation family are inhibited by oncometabolites, resulting in histone and DNA hypermethylation, respectively (Xiao *et al.* 2012, Letouze *et al.* 2013). Together, these signaling pathways promote a more aggressive pro-metastatic behavior of tumor cells (Bechmann *et al.* 2020, Morin *et al.* 2020). Additional effects of oncometabolites involve altered posttranslational modifications, including protein succinylation in *SDHx*- and *IDH1*-mutated cells (Li *et al.* 2015, Smestad *et al.* 2018) and protein succination in *FH*-mutated tumors (Yang *et al.* 2014). The latter is now being used for diagnostic purposes to detect tumors with pathogenic variants in *FH* based on 2-succinocysteine immunohistochemistry (Chen *et al.* 2014).

Metabolic changes in tumor cells with *SDHx* PV go beyond elevations of succinate, as a number of MS- and NMR-based studies have been found. Krebs cycle intermediates and amino acids, such as glutamate and aspartate, are generally lower in *SDHx*-mutated tumors compared to other PPGLs, whereas methionine and glutamine are more abundant (Imperiale *et al.* 2013, Richter *et al.* 2014, Imperiale *et al.* 2015, Richter *et al.* 2019). *In vitro* studies demonstrated that the lack of SDHx function leads to reductive carboxylation of glutamine and increased pyruvate consumption to replenish aspartate pools through pyruvate carboxylation (Cardaci *et al.* 2015, Lussey-Lepoutre *et al.* 2015). In line with these results, higher glutaminase 1 levels were reported in PPGLs with low *SDHB* expression and inhibition reduced cell growth (Sarkadi *et al.* 2020). *SDHx*-mutated tumors have lower levels of ATP/ADP/AMP compared to *VHL* and other PPGLs (Imperiale *et al.* 2013, Rao *et al.* 2013, 2015). To partially compensate for the loss of SDH or complex II from the respiratory chain, enzyme activities of complexes I, III, and IV were increased compared to sporadic *RET* and *NF1* tumors (Rao *et al.* 2013). Especially complex I was shown to be important for the oxidation of NADH to support continued Krebs cycle function (Kluckova *et al.* 2020). Inhibition of complex I resulted in reduced tumor growth in a preclinical model (Moog *et al.* 2022). Contrary to these findings, another study suggested that loss of SDH and complex I are required to recapitulate the metabolic phenotype of *SDHx*-mutant tumors (Lorendeau *et al.* 2017). These divergent results may be related to the use of cell line models originating from different tissues. Changes in respiratory chain activities can increase reactive oxygen species (ROS), which may signal oxygen insufficiency to PHDs in addition to succinate (Chandel *et al.* 2000, Guzy *et al.* 2008).

Recent data have shown that excessive succinate can lead to the disruption of Ca^2+^–calpain–cyclin-dependent kinase 5 (CDK5) signaling and aberrant activation of the protein kinase CDK5 (Gupta *et al.* 2022). Aberrant CDK5 initiates a phospho-signaling cascade that inactivates energy sensing by AMP kinase through dephosphorylation of AMP kinase and creates opportunities for cellular proliferation. Finally, a metabolomic analysis of PPGL tissues has found an increase in the polyamine pathway in *SDHx*-mutated tumors (Rai *et al.* 2020). The diamine putrescine and the polyamines spermidine and spermine are small organic cations integral to several cellular biochemical pathways and implicated in oncogenic signaling. Increased concentrations of polyamines support cell growth and inhibition suppresses xenograft growth in mice (Rai *et al.* 2020).

*FH* germline PVs are known to cause hereditary leiomyomatosis and renal cell carcinoma as well as familial non-medullary thyroid cancer (Alzahrani *et al.* 2022, Tomlinson *et al.* 2002). They also predispose to malignant PPGL and cause DNA hypermethylation similar to *SDHx* PVs (Letouze *et al.* 2013, Castro-Vega *et al.* 2014). Metabolic profiling of *FH-* and *SDHx*-mutated renal cell carcinoma identified elevated guanine pools in both tumor types, but changes in urea cycle metabolites were only present in tumors harboring *FH* PVs (Yoo *et al.* 2022). Similar results were obtained in *FH*-mutated leiomyomas (Heinonen *et al.* 2017) and are most likely similar in PPGL. High levels of the urea cycle metabolite argininosuccinate in *FH*-mutated tumors arise due to reverse activity of argininosuccinate lyase, making these cells dependent on extracellular arginine (Zheng *et al.* 2013). Additionally, NADH produced in the Krebs cycle is regenerated to NAD in the heme degradation pathway, leading to elevated bilirubin excretion and therapeutic vulnerabilities (Frezza *et al.* 2011).

Another PPGL-susceptibility gene is malate dehydrogenase 2 (*MDH2*) (Cascon *et al.* 2015). Loss of MDH2 hinders the conversion of malate to oxaloacetate in mitochondria and can cause accumulation of malate and fumarate (Ait-El-Mkadem *et al.* 2017), although metabolite accumulation in *MDH2*-mutated tumors occurs at much lower levels than in *FH*- and *SDHx*-mutated PPGLs (Richter *et al.* 2019). Similar to succinate and fumarate, malate can inhibit prolyl hydroxylation of HIFα (Pan *et al.* 2007), and global DNA hypermethylation was also detected in *MDH2*-mutated PPGLs (Cascon *et al.* 2015).

## Metabolome-guided diagnostics and future directions

Metabolomics is a useful tool for exploiting the metabolic features of PPGLs for diagnostic purposes. This section introduces a number of available methods that can aid in the metabolic stratification of PPGLs and variant classification in a subset of PPGL-susceptibility genes, thereby improving genetic diagnosis and patient management. Furthermore, methodological advances in preanalytics and the use of untargeted analytical approaches will be discussed to envisage future perspectives in the field.

Using an LC-MS/MS-based targeted quantification of Krebs cycle metabolites proved useful to identify and confirm PVs in *SDHx* and* FH* genes of PPGL samples by measuring the ratios of succinate to fumarate or fumarate to malate, respectively (Lendvai *et al.* 2014, Richter *et al.* 2014, Richter *et al.* 2019, Garrett *et al.* 2022). The same method is also useful in screening for hotspot mutations in *IDH1/2* through the detection of elevations in 2-hydroxyglutarate and especially its D-enantiomer but has less relevance in confirming the pathogenicity of these variants, since they are well described in the literature for many different tumor types and occur somatically (Dang *et al.* 2016). Beyond PPGL, the method is also applicable to other tumor types, including renal cell carcinoma and gastrointestinal stromal tumors (Kim *et al.* 2017, William *et al.* 2021).

Metabolic screening of tumors could become important, when specific therapies for metabolically dysregulated cancers with *SDHx*, *FH,* or *IDH1/2* PV become available, as targeted LC-MS/MS analysis is faster and cheaper than sequencing analyses. In this case, tumors with somatic PVs in these genes or epigenetic alterations that result in a similar phenotype are also of interest, for example *SDHC* promoter methylation in PPGL and gastrointestinal stromal tumors causing loss of *SDHC* expression and increased succinate levels (Killian *et al.* 2014, Richter *et al.* 2016). Additionally, PVs in other susceptibility genes, such as *SUCLG2* and *GOT2*, lead to elevations in succinate and might respond to similar treatments (Remacha *et al.* 2017, Hadrava Vanova *et al.* 2022). Routine metabolic screening could also aid in identifying patients with other rare variants affecting the Krebs cycle. In the future, pathologist would be best suited to extract a couple of tissue pieces per tumor of interest for further metabolite extraction, which can be accomplished from fresh-frozen or archival formalin-fixed and paraffin-embedded tissues (Richter *et al.* 2014). Depending on the tumor entity, the aim of metabolite profiling could then be to identify cancers with a possibly hereditary basis and to suggest targeted treatments when available.

Beyond succinate and fumarate, many other metabolite levels are altered in *SDHx*-mutated PPGLs and could be important diagnostically. Both LC-MS/MS and NMR studies have discovered different possible markers, including adenine nucleotides, amino acids, Krebs cycle intermediates, and polyamines (Imperiale *et al.* 2013, Rao *et al.* 2013, Richter *et al.* 2019, Rai *et al.* 2020). With the application of machine learning algorithms on LC-MS/MS tissue metabolite profiles, PPGL classification according to SDH status was improved with at least four different metabolites, especially for the more difficult-to- classify head-and-neck PGLs (Wallace *et al.* 2020). The latter contain a high percentage of non-transformed sustentacular cells that are assumed to lower succinate levels in the measured sample. For artificial intelligence to be incorporated into diagnostic procedures, larger study populations than that used in Wallace *et al.* are required to build robust algorithms without overfitting, which should be validated in prospective trials afterward (de Hond *et al.* 2022).

**Figure 3:**
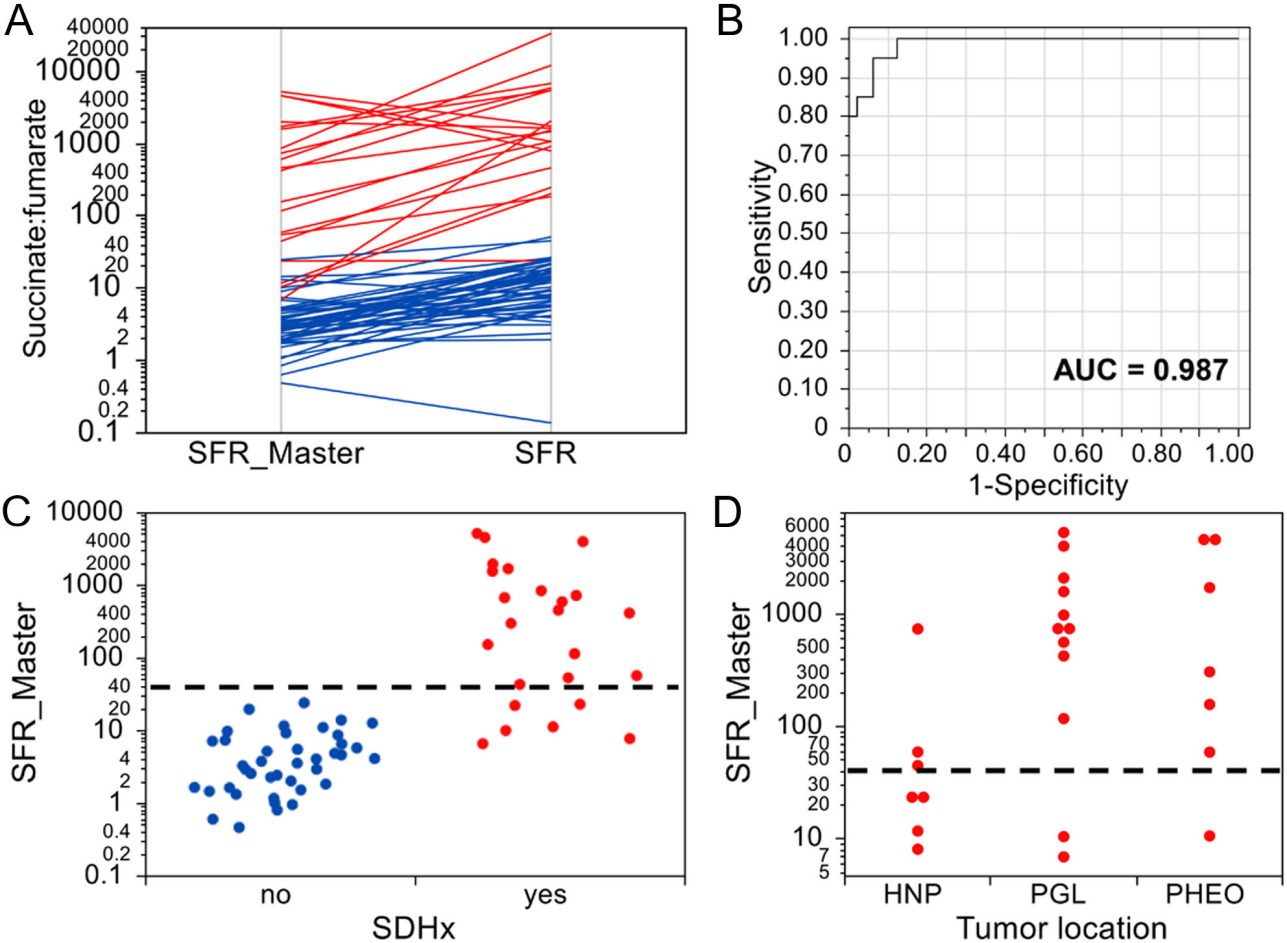
Succinate:fumarate ratios (SFR) for fresh-frozen PPGL tissues using a novel multi-purpose extraction method. (A) Comparison of the SFR between two extraction methods in the same tissue samples (*n* = 69). SFR_Master refers to the recently published method by proprietary buffer composition (Bechmann *et al.*); SFR refers to methanol extraction (Richter *et al.*). Blue, SDHx wildtype tumors; red, SDHx-mutated tumors. (B) Receiver operator characteristic curve calculations for a training set of 69 PPGLs (20 SDHx-mutated and 49 SDHx wild type) to obtain the best cutoff for SDHx-mutated samples. Cutoff was optimized to avoid false-positives (SFR_Master cutoff 40). (C) Application of the cutoff (dashed line) to a validation cohort of 76 PPGLs (6 SDHx-mutated and 70 SDHx wild type). (D) Four of seven false-negatives (training + validation set) fall into the category of head-and-neck paragangliomas (HNP). AUC, area under the curve; PHEO, pheochromocytoma; PGL, abdominal or thoracic paraganglioma.

Recently, we reported a methodological improvement of LC-MS/MS-mediated tissue metabolomics, by showing that a commercial buffer enables analysis by multiple targeted and untargeted LC-MS/MS assays, as well as RNA and protein analyses, including enzyme activities, from the same piece of tissue (Bechmann *et al.* 2021). Other groups have also published on simultaneous tissue extraction methods for multi-omics focusing on proteins or RNA in combination with metabolites and lipids (Coman *et al.* 2016, Leuthold *et al.* 2018, Kang *et al.* 2021). Such developments are extremely important for procedures that integrate information from different assays to overcome effects due to tumor heterogeneity. Additionally, procedures that require minimal tissue input are critical for research of rare tumor entities, such as PPGLs, and for animal studies. We further validated our new multi-omics extraction method by comparing the resulting succinate:fumarate ratio (SFR) to those generated with the previously employed methanol extraction (Fig. 3). The SFR is generally lower with the new extraction, but PPGLs with *SDHx* mutation are mostly identified, resulting in an area under the curve of 0.987. An SFR cutoff of 40 for the multi-purpose extraction classified all *SDHx*-wildtype PPGLs correctly, but three of six *SDHx*-mutated tumors of a validation set were false-negatives. From the seven false-negatives that were present in the training and the validation sets, four belonged to the group of head-and-neck PGLs, two were PGLs, and one was a PHEO. Selection of an appropriate piece of tissue is similar to the methanol extraction method paramount to receiving the right classification. Hence, parallel testing of at least two different tissue pieces and selection by a pathologist might increase the reliability of classifications. With such a multi-purpose method, additional analyses looking at the expression of specific chromaffin markers vs those of sustentacular cells could be helpful in assessing the suitability of the tissue piece.

In some cases, an operation is not possible or not indicated, for example for slow-growing head-and-neck PGLs, where sometimes a wait-and-scan approach is recommended, less invasive methods for the assessment of metabolite profiles are required (Jansen *et al.* 2017). One such method is NMR with respect to hydrogen-1 nuclei, also referred to as proton magnetic resonance spectroscopy (^1^H-MRS), in combination with MRI. It was demonstrated that this technique detects succinate elevations exclusively in *SDHx*-mutated PPGLs (Varoquaux *et al.* 2015, Lussey-Lepoutre *et al.* 2016). Larger tumors give higher quality spectra, whereas small head-and-neck PGLs, for example of the jugular region, are more difficult to analyze due to current detection limits of instruments. Further limitations include tumors with hemorrhagic or necrotic spots. A study with 49 patients calculated a sensitivity of 87% and specificity of 100% for succinate detection in *SDHx*-mutated PPGLs (Lussey-Lepoutre *et al.* 2020). Since operation costs are high and radiological expertise and experience is required, this technique is most appropriate for specialized PPGL clinical centers. In the future, 7-tesla MRIs might improve post-processing of spectra to increase sensitivity.

Whereas ^1^H-MRS is useful to identify *SDHx*-mutated tumors *in vivo*, it is not of course applicable to *SDHx* PV carriers who have not yet developed tumors. A recent publication reported that carriers of *SDHB* PV have elevated levels of serum succinate (Lamy *et al.* 2022). Patients with *SDHC* or *SDHD* PV, of which very few were tested, had smaller increases of succinate. To validate this method for diagnostic use in PV carriers, reference intervals from a large group of healthy individuals of different ages and body mass index should be generated, since succinate elevations in plasma were also linked to the risk of cardiovascular disease and obesity (Serena *et al.* 2018, Osuna-Prieto *et al.* 2021). Furthermore, serum succinate correlated with the metastatic load of patients with tumors due to an *SDHB* PV and can be used as a biomarker to monitor treatment success and recurrence. Since blood samples are easily obtainable, they are the preferred diagnostic materials and hold a lot of potential for the future. As cell line models of PPGL with loss of about half of the SDH protein show only moderate succinate increases, it is rather surprising that succinate differences are measurable in the serum (Richter *et al.* 2018). Further studies will show whether other metabolites improve the classification of *SDHx* mutation carriers in the blood and whether this method is applicable for patients with other germline PV, such as *FH*. Table 1 summarizes all currently available methods for metabolic assessment of PPGLs with variants in *SDHx* genes.

**Table 1:** Overview of analytical methods for the measurement of succinate for functional validation of variants in *SDHx* genes.

Material	Platform	Advantages	Disadvantages
Tissue *ex vivo*	LC-MS/MS	–Validated on a large patient cohort –Known to be applicable also for other rare gene mutations (*FH*, *IDH1/2*) –Instrument is widely available (same as metanephrine analytic) –Fast and cheap	–OP necessary –Not applicable for *SDHx* mutation carriers without tumor
*In vivo* tumor	^1^H-MRS + MRI	–Pre-OP measurement possible	–Not widely available instrumentation –Expensive –Not applicable for *SDHx* mutation carriers without tumor
Serum	LC-MS/MS	–No OP necessary –Sampling most comfortable for patient –Possibility of evaluation of *SDHB* mutation carriers –Follow-up of treatment efficacy –Fast and cheap	–Only shown in one cohort for patients with *SDHB* mutations

^1^H-MRS, proton magnetic resonance spectroscopy; LC-MS/MS, liquid chromatography with mass spectrometry; MRI, magnetic resonance imaging; NMR, nuclear resonance spectroscopy; OP, operation.

**Figure 4:**
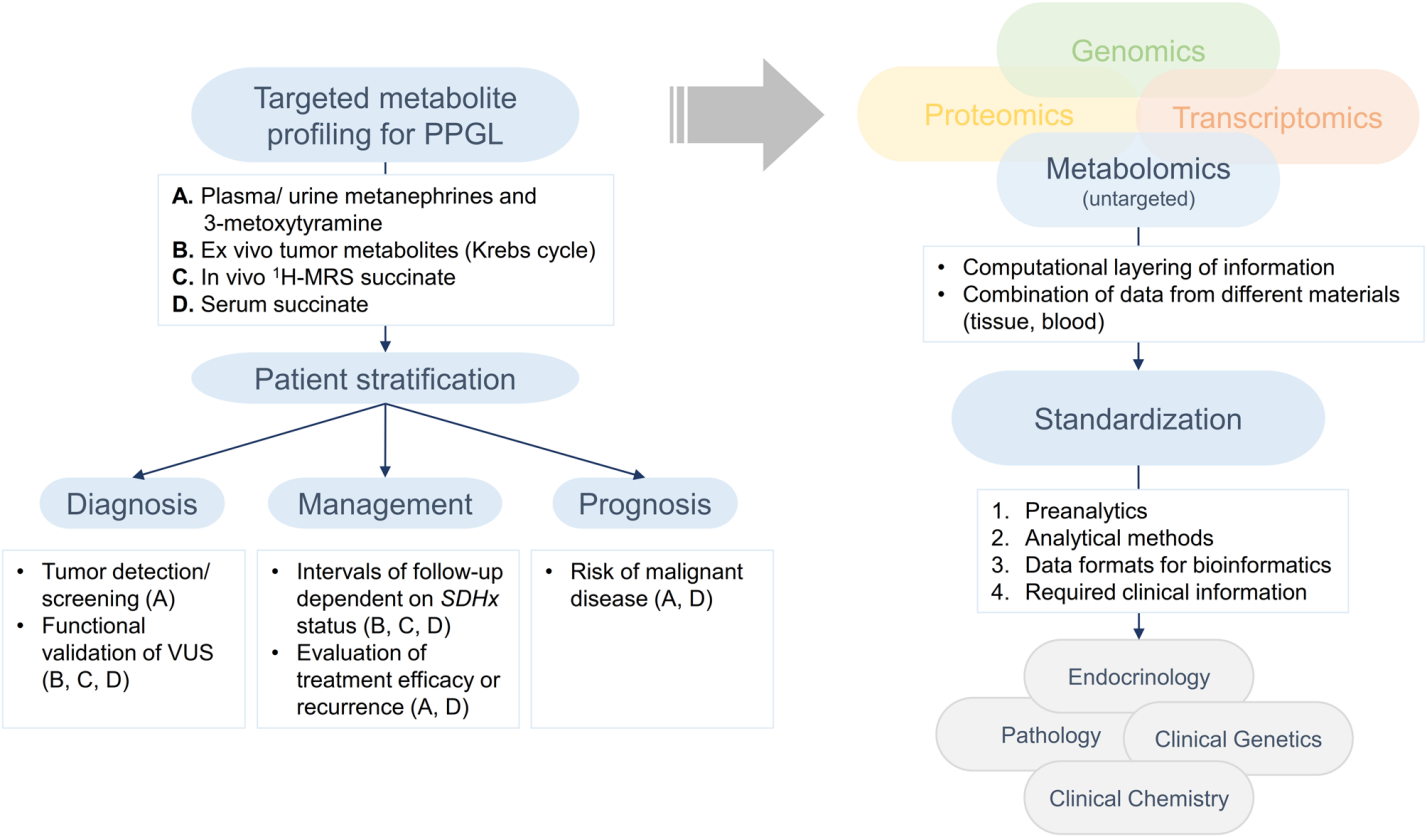
Metabolic assessment of PPGL now (left) and future perspectives (right). ^1^H-MRS, proton magnetic resonance spectroscopy; VUS, genetic variants of unknown significance.

Diagnostic methods discussed herein for the metabolic assessment of PPGLs are all targeted analyses focusing either on metanephrines or on succinate as the main metabolites and aid in stratifying patients suffering from PPGLs (Fig. 4). The presented techniques of metabolome characterization are not only important for diagnosis, which includes tumor screening by metanephrines and functional validation of variants of unknown significance in *SDHx* genes, but they also guide patient management and prognosis. Depending on germline *SDHx* status, certain follow-up times for patients with PPGL or mutation carriers are recommended (Amar *et al.* 2021). In addition, serum succinate analysis has the potential as a marker for treatment efficacy in *SDHx*-mutated PPGLs, and plasma 3-methoxytyramine has prognostic value for risk assessment of metastatic disease. Recently, an untargeted metabolomics study using MALDI-MS imaging of formalin-fixed paraffin-embedded PPGL specimens indicated that the abundance of kynurenine pathway metabolites that originate from tryptophan catabolism was significantly lower in PPGLs with activation of pseudohypoxia pathways compared to kinase-driven PPGLs (Murakami *et al.* 2021). Lower abundance of the metabolite xanthurenic acid was associated with shorter metastasis-free survival and identified as a risk factor for metastasis independent of the genetic status. Although not yet validated in other cohorts or with targeted MS assays, xanthurenic acid could become another important biomarker for the management of PPGL patients.

In the future, metabolomic assessment of patients with PPGL, in the tumor tissue and blood, will most likely move toward broader metabolic signatures using bigger targeted panels or untargeted approaches that will be combined with genomic and possibly transcriptomic or proteomic information. In this respect, the methods for efficient extraction of different molecules from the same piece of tumor become very important (Bechmann *et al.* 2021). NMR-based untargeted metabolomics studies have so far only been performed on a very limited number of PPGL tissues. The technique was useful in classifying PPGLs based on their mutational backgrounds, which applied not only to *SDHx*-mutated tumors but also to *VHL* and *RET* mutations (Imperiale *et al.* 2015, Rao *et al.* 2015). Metabolites that may help to better classify *SDHx-* and *FH*-mutated PPGLs are guanine, acylcarnitines for *SDHx* and urea cycle metabolites like argininosuccinate for *FH* mutant tumors. Although data have so far been presented only for renal cell carcinomas, it is highly likely that these features are also present in PPGLs (Yoo *et al.* 2022). Omics setups that recognize patterns rather than isolated alterations could further improve patient stratification, for example according to risk of metastasis or recurrence and eligibility for targeted therapies. Two recent studies of plasma from patients with PPGL used targeted LC-MS/MS with an extended panel of over 100 metabolites. Unfortunately, low patient numbers and high interindividual differences may have prevented the identification of suitable signatures for PPGL diagnosis, but it was demonstrated that the metabolic aberrations that occurred with PPGL were reversed after surgery (Erlic *et al.* 2019, Marz *et al.* 2021).

In order to gather meaningful data suitable for computational analysis, a number of critical steps have to be taken, which are especially important when different medical and analytical centers are involved in data collection and processing. Preanalytical procedures of sample collection, handling, and storage have to be standardized to avoid strong confounding factors that might mask underlying effects. A recent study using NMR metabolomics in plasma reported sample age and origin as major confounders (Bliziotis *et al.* 2022). Furthermore, analytical methods used in different centers have to be harmonized to further avoid confounders and ensure reproducibility, as it was described for LC-MS/MS-based plasma metanephrine measurements (Peitzsch *et al.* 2021). Data formats and outputs should be compatible, and all required clinical information has to be provided in appropriate data sheets. Furthermore, ethical considerations have to be resolved to avoid misuse of omics data against the patient’s consent. The setup of such an omics-based diagnostic approach requires intense communication between medical disciplines and clinical centers to establish the necessary infrastructure. The European cooperation in science and technology funded the harmonization of clinical care and research on adrenal tumors in European countries (CA20122), providing the first step into a future of standardized medical care involving multi-omics-based diagnostic for PPGL. Due to the high costs connected to the computational requirements for bioinformatics analyses and data storage, not all PPGL patients might be examined by multi-omics, but selected cases with inoperable tumors or risk of metastases could benefit. Additionally, rare gene mutations might be identified based on unusual omics patterns.

## Therapeutic strategies for PPGLs and oncometabolite-driven tumors

The mainstay for treatment in PPGL is surgical with preoperative antihypertensive treatment with alpha receptor antagonists a few weeks prior to tumor resection. If a patient has rapidly growing metastatic disease, the patient will have to undergo conventional chemotherapy with cyclophosphamide, vincristine, and dacarbazine that interfere with DNA replication and mitosis (Jawed *et al.* 2018). Another option may be the alkylating agent temozolomide alone or combined with the antimetabolite capecitabine, which inhibits DNA synthesis (Hadoux *et al.* 2014, Perez *et al.* 2022). For patients that have more slowly growing tumors, radiopharmaceutical agents, such as ^131^I-metaiodobenzylguanidine targeting the norepinephrine transport system or peptide receptor radionuclide therapy (PRRT) taking advantage of somatostatin receptor type 2 expression, can be an option (Pryma *et al.* 2019, Vyakaranam *et al.* 2019). Despite these available therapies, a complete cure for metastatic PPGL is still not available. Investigators have turned now toward specific alterations in tumor signaling and metabolism to identify new treatment targets that may help to achieve a durable and satisfactory response to aggressive disease.

Since roughly 50% of metastatic PPGLs carry PVs in *SDHB* (Pamporaki *et al.* 2022), oncometabolite-driven signaling pathways and metabolic vulnerabilities are a focal point of new therapeutic approaches (Fig. 2). Recent research has uncovered that the oncometabolites fumarate and succinate suppress the homologous recombination DNA repair pathway responsible for maintaining genomic integrity through the inhibition of lysine demethylases KDM4A/B (Sulkowski *et al.* 2018). This observation renders affected tumor cells vulnerable to targeting with poly-(ADP-ribose) polymerase (PARP) inhibitors. Mouse PPGL models confirmed that a combination of temozolomide and PARP inhibitor reduced metastatic lesions and improved overall survival (Pang *et al.* 2018). Combination of these two agents is currently evaluated in a phase II clinical study (NCT04394858). Additional opportunities may arise with agents targeting DNA or histone methylation (Pang *et al.* 2019); however, since DNA hypermethylation alone is not sufficient to drive mesenchymal transition, monotherapy may not be effective against metastatic PPGL (Morin *et al.* 2020). Instead, it may be useful as adjuvant therapy supporting PRRT (Ullrich *et al.* 2023).

Further opportunities have emerged with the understanding that increases in unhydroxylated HIF through either oncometabolite mediated inhibition of PHDs, loss of VHL, or mutations in the hydroxylation domain of *EPAS1* activate vascular endothelial growth factor, a driver of angiogenesis. Inhibiting the angiogenic pathway with the receptor tyrosine kinase inhibitor sunitinib showed moderate tumor regression and stability with treatment (Joshua *et al.* 2009). Newer data from the First International Randomized Study in Malignant Progressive Pheochromocytoma and Paragangliomas (FIRSTMAPPP, NCT01371201) indicate that 35.9% of patients with progressive malignant PPGL treated with sunitinib achieved the primary endpoint of progression-free survival at 12 months (Baudin *et al.* 2021). The median progression-free survival was 8.9 months with sunitinib vs 3.6 months in the placebo arm. Another approach is based on the inhibition of HIF2A by the specific inhibitor belzutifan, which disrupts binding to its heterodimerization partner aryl hydrocarbon receptor nuclear translocator (Ren *et al.* 2022). Belzutifan was approved last year for cancers associated with VHL disease but may also show benefits in patients with PPGLs caused by *EPAS1* (Kamihara *et al.* 2021) and other mutations causing a pseudohypoxia phenotype. In preclinical models for *SDHx*-mutated PPGL, an earlier generation HIF2A inhibitor or belzutifan did not show antitumor activity, while sunitinib induced a significant reduction of tumor volume (Bechmann *et al.* 2020, Moog *et al.* 2022).

Specifically in *SDHB*-mutated PPGLs and cell line models, intracellular iron accumulation leads to the generation of oxidative stress and increased mitochondrial ROS (Liu *et al.* 2020*b*, Goncalves *et al.* 2021). As a result, treatment with ascorbic acid may be a promising strategy but requires clinical testing and investigations into administration and dosage. In another approach, researchers have targeted nuclear factor erythroid 2-related factor 2-guided glutathione synthesis in preclinical models of *SDHB*-mutated PPGL (Liu *et al.* 2020*a*).

Another promising target that has been investigated preclinically is the polyamine pathway. Application of the polyamine analogue *N* ^1^ **,** *N* ^11^-bis(ethyl) norspermine (BENSpm/DENSPM) induced growth arrest in a xenograft mouse model and was also highly effective in cells with loss of *SDHB* (Rai *et al.* 2020). Phase 1 clinical trials in other cancers demonstrated that DENSPM can be administered with minimal toxicities, but single therapy showed no objective disease responses, indicating the need for identifying effective drug combinations (Streiff & Bender 2001, Hahm *et al.* 2002). With the recognition of elevated succinate causing aberrant activation of CDK5, the anti-CDK5 inhibitor MRT3-007 was shown to suppress tumor growth *in vitro* as well as in xenograft mouse models with *SDHB* knockout (Gupta *et al.* 2022). Other approaches to inhibit aberrations in *SDHx*-mutated PPGL that might warrant further exploration include inhibition of glutaminase, respiratory complex I with IACS-010759, or pyruvate carboxylase (Cardaci *et al.* 2015, Sarkadi *et al.* 2020, Moog *et al.* 2022). For the latter, unfortunately, no promising inhibitors have been identified. A phase I study of glutaminase inhibitor CB-839 in patients with *SDHx*-, *FH*- or *IDHx*-mutated tumors completed recruitment in 2022 (NCT02071862). Further opportunities may arise from investigations of synthetically lethal pathway aberrations, as demonstrated by the loss of *FH* in combination with inhibition of heme oxygenation (Frezza *et al.* 2011).

Other metabolic drugs, including antidiabetic agent metformin, pyruvate dehydrogenase inhibitor dichloroacetate, and peroxisome proliferator-activated receptor α inhibitor GW6471, reduce cell viability and clonogenicity *in vitro*; however, whether these treatments are specific to cells with *SDHx* mutations is unclear (Florio *et al.* 2018). Interestingly, metformin lowers the migratory capacity specifically in *SDHB*-silenced cells cocultured with fibroblasts but not in wildtype tumor cells (Martinelli *et al.* 2022). Since metformin activates AMP kinase by phosphorylation, it may counteract the inhibitory effect of succinate-dependent CDK5 activity on AMP kinase (Zhou *et al.* 2001, Gupta *et al.* 2022).

Different combinations of established cancer therapies and new experimental agents should be investigated in the future, focusing on targeting two or more pathways important for tumor maintenance or metastasis at the same time. These combinations can be tailored to the specific mutations and clinical presentations of the individual patient. Metabolomics of tissue samples or liquid biopsies could be a very useful tool for accompanying clinical trials to identify biomarkers for treatment responders or non-responders. Additionally, metabolic profiles of these patients might give new clues about which further treatments are promising.

## Conclusions

Cellular metabolism is key to all processes of life, and especially cancer cells have developed a multitude of strategies to support proliferation, ensure survival by adapting to changing environments, and escape anti-cancer treatments. Exploring metabolic alterations by targeted or untargeted approaches resulted in clinical biomarkers for diagnosis of PPGLs in urine or blood and of subforms with mutations in Krebs cycle genes. Metabolomic information from tumors have provided clues for potential treatment targets. These include pathways affecting angiogenesis, ROS, DNA repair, polyamines, and kinase cascades. With further advances, tailored therapies for subgroups of PPGLs, for example those with specific gene mutations and transcriptomic or metabolomic signatures, will become available. Quantification of the oncometabolite succinate in the blood will be a useful tool for following therapy effects in *SDHB*-mutated metastatic PPGL. Metabolomics will play an important role together with the other omics disciplines in paving the way towards a better future for patients with metastatic PPGL.

## Declaration of interest

The authors declare that there is no conflict of interest that could be perceived as prejudicing the impartiality of the research reported.

## Funding

SR and NB are supported by the Deutsche Forschungsgemeinschaft (DFG, German Research Foundation) project number: 314061271 – TRR 205. HKG is supported by the NIH R21 TR003044-01A1.

## References

[bib1] Ait-El-Mkadem S Dayem-Quere M Gusic M Chaussenot A Bannwarth S Francois B Genin EC Fragaki K Volker-Touw CLM Vasnier C, 2017 Mutations in MDH2, encoding a Krebs cycle enzyme, cause early-onset severe encephalopathy. American Journal of Human Genetics 100 151–159. (10.1016/j.ajhg.2016.11.014) 27989324 PMC5223029

[bib2] Alseekh S Aharoni A Brotman Y Contrepois K D’auria J Ewald J C Ewald J Fraser PD Giavalisco P Hall RD, 2021 Mass spectrometry-based metabolomics: a guide for annotation, quantification and best reporting practices. Nature Methods 18 747–756. (10.1038/s41592-021-01197-1) 34239102 PMC8592384

[bib3] Alzahrani AS Alswailem M Alghamdi B & Al-Hindi H 2022 Fumarate hydratase is a novel gene for familial non-medullary thyroid cancer. Journal of Clinical Endocrinology and Metabolism 107 2539–2544. (10.1210/clinem/dgac386) 35751867

[bib4] Amar L Pacak K Steichen O Akker SA Aylwin SJB Baudin E Buffet A Burnichon N Clifton-Bligh RJ Dahia PLM, 2021 International consensus on initial screening and follow-up of asymptomatic SDHx mutation carriers. Nature Reviews. Endocrinology 17 435–444. (10.1038/s41574-021-00492-3) PMC8205850 34021277

[bib5] Baudin E Goichot B Berruti A Hadoux J Moalla S Laboureau S Noelting S De La Fouchardière C Kienitz T Deutschbein T, 2021 567O First International Randomized Study in Malignant Progressive pheochromocytoma and paragangliomas (FIRSTMAPPP): an academic double-blind trial investigating sunitinib. Annals of Oncology 32 S621. (10.1016/j.annonc.2021.08.702)

[bib6] Bechmann N Moskopp ML Ullrich M Calsina B Wallace PW Richter S Friedemann M Langton K Fliedner SMJ Timmers HJLM, 2020 HIF2alpha supports pro-metastatic behavior in pheochromocytomas/paragangliomas. Endocrine-Related Cancer 27 625–640. (10.1530/ERC-20-0205) 33112842

[bib7] Bechmann N Watts D Steenblock C Wallace PW Schurmann A Bornstein SR Wielockx B Eisenhofer G & Peitzsch M 2021 Adrenal hormone interactions and metabolism: a single sample multi-omics approach. Hormone and Metabolic Research 53 326–334. (10.1055/a-1440-0278) 33902135 PMC8105089

[bib8] Bliziotis NG Kluijtmans LAJ Tinnevelt GH Reel P Reel S Langton K Robledo M Pamporaki C Pecori A Van Kralingen J, 2022 Preanalytical pitfalls in untargeted plasma nuclear magnetic resonance metabolomics of endocrine hypertension. Metabolites 12. (10.3390/metabo12080679) PMC9394285 35893246

[bib9] Cardaci S Zheng L Mackay G Van Den Broek NJ Mackenzie ED Nixon C Stevenson D Tumanov S Bulusu V Kamphorst JJ, 2015 Pyruvate carboxylation enables growth of SDH-deficient cells by supporting aspartate biosynthesis. Nature Cell Biology 17 1317–1326. (10.1038/ncb3233) 26302408 PMC4591470

[bib10] Cascon A Comino-Mendez I Curras-Freixes M De Cubas AA Contreras L Richter S Peitzsch M Mancikova V Inglada-Perez L Perez-Barrios A, 2015 Whole-exome sequencing identifies MDH2 as a new familial paraganglioma gene. Journal of the National Cancer Institute 107 djv053. (10.1093/jnci/djv053) 25766404

[bib11] Castro-Vega LJ Buffet A De Cubas AA Cascon A Menara M Khalifa E Amar L Azriel S Bourdeau I Chabre O, 2014 Germline mutations in FH confer predisposition to malignant pheochromocytomas and paragangliomas. Human Molecular Genetics 23 2440–2446. (10.1093/hmg/ddt639) 24334767

[bib12] Chandel NS Mcclintock DS Feliciano CE Wood TM Melendez JA Rodriguez AM & Schumacker PT 2000 Reactive oxygen species generated at mitochondrial complex III stabilize hypoxia-inducible factor-1alpha during hypoxia: a mechanism of O2 sensing. Journal of Biological Chemistry 275 25130–25138. (10.1074/jbc.M001914200) 10833514

[bib13] Chen YB Brannon AR Toubaji A Dudas ME Won HH Al-Ahmadie HA Fine SW Gopalan A Frizzell N Voss MH, 2014 Hereditary leiomyomatosis and renal cell carcinoma syndrome-associated renal cancer: recognition of the syndrome by pathologic features and the utility of detecting aberrant succination by immunohistochemistry. American Journal of Surgical Pathology 38 627–637. (10.1097/PAS.0000000000000163) 24441663 PMC3984629

[bib14] Coman C Solari FA Hentschel A Sickmann A Zahedi RP & Ahrends R 2016 Simultaneous metabolite, protein, lipid extraction (Simplex): a combinatorial multimolecular omics approach for systems biology. Molecular and Cellular Proteomics 15 1453–1466. (10.1074/mcp.M115.053702) 26814187 PMC4824867

[bib15] Curras-Freixes M Pineiro-Yanez E Montero-Conde C Apellaniz-Ruiz M Calsina B Mancikova V Remacha L Richter S Ercolino T Rogowski-Lehmann N, 2017 PheoSeq: a targeted next-generation sequencing assay for pheochromocytoma and paraganglioma diagnostics. Journal of Molecular Diagnostics 19 575–588. (10.1016/j.jmoldx.2017.04.009) PMC5500830 28552549

[bib16] Dang L Yen K & Attar EC 2016 IDH mutations in cancer and progress toward development of targeted therapeutics. Annals of Oncology 27 599–608. (10.1093/annonc/mdw013) 27005468

[bib17] Davidoff DF Benn DE Field M Crook A Robinson BG Tucker K De Abreu Lourenco R Burgess JR & Clifton-Bligh RJ 2022 Surveillance improves outcomes for carriers of SDHB pathogenic variants: a multicenter study. Journal of Clinical Endocrinology and Metabolism 107 e1907–e1916. (10.1210/clinem/dgac019) PMC9016424 35037935

[bib18] de Hond AAH Leeuwenberg AM Hooft L Kant IMJ Nijman SWJ Van Os HJA Aardoom JJ Debray TPA Schuit E Van Smeden M, 2022 Guidelines and quality criteria for artificial intelligence-based prediction models in healthcare: a scoping review. npj Digital Medicine 5 2. (10.1038/s41746-021-00549-7) PMC8748878 35013569

[bib19] Eisenhofer G Klink B Richter S Lenders JW & Robledo M 2017 Metabologenomics of phaeochromocytoma and paraganglioma: an integrated approach for personalised biochemical and genetic testing. Clinical Biochemist. Reviews 38 69–100. 29332973 PMC5759086

[bib20] Eisenhofer G Kopin IJ & Goldstein DS 2004 Catecholamine metabolism: a contemporary view with implications for physiology and medicine. Pharmacological Reviews 56 331–349. (10.1124/pr.56.3.1) 15317907

[bib21] Eisenhofer G Lenders JW Siegert G Bornstein SR Friberg P Milosevic D Mannelli M Linehan WM Adams K Timmers HJ, 2012 Plasma methoxytyramine: a novel biomarker of metastatic pheochromocytoma and paraganglioma in relation to established risk factors of tumour size, location and SDHB mutation status. European Journal of Cancer 48 1739–1749. (10.1016/j.ejca.2011.07.016) 22036874 PMC3372624

[bib22] Eisenhofer G & Peitzsch M 2014 Laboratory evaluation of pheochromocytoma and paraganglioma. Clinical Chemistry 60 1486–1499. (10.1373/clinchem.2014.224832) 25332315

[bib23] Erlic Z Kurlbaum M Deutschbein T Nolting S Prejbisz A Timmers H Richter S Prehn C Weismann D Adamski J, 2019 Metabolic impact of pheochromocytoma/paraganglioma: targeted metabolomics in patients before and after tumor removal. European Journal of Endocrinology 181 647–657. (10.1530/EJE-19-0589) 31614337

[bib24] Fishbein L Leshchiner I Walter V Danilova L Robertson AG Johnson AR Lichtenberg TM Murray BA Ghayee HK Else T, 2017 Comprehensive molecular characterization of pheochromocytoma and paraganglioma. Cancer Cell 31 181–193. (10.1016/j.ccell.2017.01.001) 28162975 PMC5643159

[bib25] Florio R De Lellis L Veschi S Verginelli F Di Giacomo V Gallorini M Perconti S Sanna M Mariani-Costantini R Natale A, 2018 Effects of dichloroacetate as single agent or in combination with GW6471 and metformin in paraganglioma cells. Scientific Reports 8 13610. (10.1038/s41598-018-31797-5) PMC6134030 30206358

[bib26] Frezza C Zheng L Folger O Rajagopalan KN Mackenzie ED Jerby L Micaroni M Chaneton B Adam J Hedley A, 2011 Haem oxygenase is synthetically lethal with the tumour suppressor fumarate hydratase. Nature 477 225–228. (10.1038/nature10363) 21849978

[bib27] Garrett A Loveday C King L Butler S Robinson R Horton C Yussuf A Choi S Torr B Durkie M, 2022 Quantifying evidence toward pathogenicity for rare phenotypes: the case of succinate dehydrogenase genes, SDHB and SDHD. Genetics in Medicine 24 41–50. (10.1016/j.gim.2021.08.004) 34906457 PMC8759765

[bib28] Gimenez-Roqueplo AP Favier J Rustin P Rieubland C Crespin M Nau V Khau Van Kien P Corvol P Plouin PF Jeunemaitre X, 2003 Mutations in the SDHB gene are associated with extra-adrenal and/or malignant phaeochromocytomas. Cancer Research 63 5615–5621. 14500403

[bib29] Goncalves J Moog S Morin A Gentric G Muller S Morrell AP Kluckova K Stewart TJ Andoniadou CL Lussey-Lepoutre C, 2021 Loss of SDHB promotes dysregulated iron homeostasis, oxidative stress, and sensitivity to ascorbate. Cancer Research 81 3480–3494. (10.1158/0008-5472.CAN-20-2936) 34127497 PMC7616967

[bib30] Gupta P Strange K Telange R Guo A Hatch H Sobh A Elie J Carter AM Totenhagen J Tan C, 2022 Genetic impairment of succinate metabolism disrupts bioenergetic sensing in adrenal neuroendocrine cancer. Cell Reports 40 111218. (10.1016/j.celrep.2022.111218) PMC9822535 35977518

[bib31] Guzy RD Sharma B Bell E Chandel NS & Schumacker PT 2008 Loss of the SdhB, but not the SdhA, subunit of complex II triggers reactive oxygen species-dependent hypoxia-inducible factor activation and tumorigenesis. Molecular and Cellular Biology 28 718–731. (10.1128/MCB.01338-07) 17967865 PMC2223429

[bib32] Hadoux J Favier J Scoazec JY Leboulleux S Al Ghuzlan A Caramella C Déandreis D Borget I Loriot C Chougnet C, 2014 SDHB mutations are associated with response to temozolomide in patients with metastatic pheochromocytoma or paraganglioma. International Journal of Cancer 135 2711–2720. (10.1002/ijc.28913) 24752622

[bib33] Hadrava Vanova K Pang Y Krobova L Kraus M Nahacka Z Boukalova S Pack SD Zobalova R Zhu J Huynh TT, 2022 Germline SUCLG2 variants in patients with pheochromocytoma and paraganglioma. Journal of the National Cancer Institute 114 130–138. (10.1093/jnci/djab158) 34415331 PMC8755484

[bib34] Hahm HA Ettinger DS Bowling K Hoker B Chen TL Zabelina Y & Casero RA 2002 Phase I study of N(1),N(11)-diethylnorspermine in patients with non-small cell lung cancer. Clinical Cancer Research 8 684–690. 11895896

[bib35] Heinonen HR Mehine M Makinen N Pasanen A Pitkanen E Karhu A Sarvilinna NS Sjoberg J Heikinheimo O Butzow R, 2017 Global metabolomic profiling of uterine leiomyomas. British Journal of Cancer 117 1855–1864. (10.1038/bjc.2017.361) 29073636 PMC5729474

[bib37] Imperiale A Moussallieh FM Sebag F Brunaud L Barlier A Elbayed K Bachellier P Goichot B Pacak K Namer IJ, 2013 A new specific succinate-glutamate metabolomic hallmark in sdhx-related paragangliomas. PLoS One 8 e80539. (10.1371/journal.pone.0080539) PMC3842321 24312232

[bib36] Imperiale A Moussallieh FM Roche P Battini S Cicek AE Sebag F Brunaud L Barlier A Elbayed K Loundou A, 2015 Metabolome profiling by HRMAS NMR spectroscopy of pheochromocytomas and paragangliomas detects SDH deficiency: clinical and pathophysiological implications. Neoplasia 17 55–65. (10.1016/j.neo.2014.10.010) 25622899 PMC4309730

[bib38] Janeway KA Kim SY Lodish M Nosé V Rustin P Gaal J Dahia PL Liegl B Ball ER Raygada M, 2011 Defects in succinate dehydrogenase in gastrointestinal stromal tumors lacking KIT and PDGFRA mutations. PNAS 108 314–318. (10.1073/pnas.1009199108) 21173220 PMC3017134

[bib39] Jansen TTG Timmers HJLM Marres HAM & Kunst HPM 2017 Feasibility of a wait-and-scan period as initial management strategy for head and neck paraganglioma. Head and Neck 39 2088–2094. (10.1002/hed.24871) 28691354

[bib40] Jawed I Velarde M Darr R Wolf KI Adams K Venkatesan AM Balasubramaniam S Poruchynsky MS Reynolds JC Pacak K, 2018 Continued tumor reduction of metastatic pheochromocytoma/paraganglioma harboring succinate dehydrogenase Subunit B mutations with cyclical chemotherapy. Cellular and Molecular Neurobiology 38 1099–1106. (10.1007/s10571-018-0579-4) 29623478 PMC5976545

[bib41] Jhawar S Arakawa Y Kumar S Varghese D Kim YS Roper N Elloumi F Pommier Y Pacak K & Del Rivero J 2022 New insights on the genetics of pheochromocytoma and paraganglioma and its clinical implications. Cancers (Basel) 14 594. (10.3390/cancers14030594) 35158861 PMC8833412

[bib42] Joshua AM Ezzat S Asa SL Evans A Broom R Freeman M & Knox JJ 2009 Rationale and evidence for sunitinib in the treatment of malignant paraganglioma/pheochromocytoma. Journal of Clinical Endocrinology and Metabolism 94 5–9. (10.1210/jc.2008-1836) 19001511

[bib43] Kamihara J Hamilton KV Pollard JA Clinton CM Madden JA Lin J Imamovic A Wall CB Wassner AJ Weil BR, 2021 Belzutifan, a potent HIF2alpha inhibitor, in the Pacak-Zhuang syndrome. New England Journal of Medicine 385 2059–2065. (10.1056/NEJMoa2110051) 34818480 PMC11245359

[bib44] Kang J David L Li Y Cang J & Chen S 2021 Three-in-one simultaneous extraction of proteins, metabolites and lipids for multi-omics. Frontiers in Genetics 12 635971. (10.3389/fgene.2021.635971) PMC8082496 33936167

[bib45] Kes MMG Van Den Bossche J Griffioen AW & Huijbers EJM 2020 Oncometabolites lactate and succinate drive pro-angiogenic macrophage response in tumors. Biochimica et Biophysica Acta. Reviews on Cancer 1874 188427. (10.1016/j.bbcan.2020.188427) 32961257

[bib46] Killian JK Miettinen M Walker RL Wang Y Zhu YJ Waterfall JJ Noyes N Retnakumar P Yang Z Smith WI, 2014 Recurrent epimutation of SDHC in gastrointestinal stromal tumors. Science Translational Medicine 6 268ra177. (10.1126/scitranslmed.3009961) PMC7670881 25540324

[bib47] Kim E Wright MJ Sioson L Novos T Gill AJ Benn DE White C Dwight T & Clifton-Bligh RJ 2017 Utility of the succinate: fumarate ratio for assessing SDH dysfunction in different tumor types. Molecular Genetics and Metabolism Reports 10 45–49. (10.1016/j.ymgmr.2016.12.006) 28070496 PMC5219629

[bib48] Kluckova K Thakker A Vettore L Escribano-Gonzalez C Hindshaw RL Tearle JLE Goncalves J Kaul B Lavery GG Favier J, 2020 Succinate dehydrogenase deficiency in a chromaffin cell model retains metabolic fitness through the maintenance of mitochondrial NADH oxidoreductase function. FASEB Journal 34 303–315. (10.1096/fj.201901456R) 31914648

[bib49] Lamy C Tissot H Faron M Baudin E Lamartina L Pradon C Al Ghuzlan A Leboulleux S Perfettini JL Paci A, 2022 Succinate: a serum biomarker of SDHB mutated paragangliomas and pheochromocytomas. Journal of Clinical Endocrinology and Metabolism 107 2801–2810. (10.1210/clinem/dgac474) 35948272

[bib50] Lenders JW Duh QY Eisenhofer G Gimenez-Roqueplo AP Grebe SK Murad MH Naruse M Pacak K Young WF & Endocrine Society 2014 Pheochromocytoma and paraganglioma: an endocrine society clinical practice guideline. Journal of Clinical Endocrinology and Metabolism 99 1915–1942. (10.1210/jc.2014-1498) 24893135

[bib51] Lendvai N Pawlosky R Bullova P Eisenhofer G Patocs A Veech RL & Pacak K 2014 Succinate-to-fumarate ratio as a new metabolic marker to detect the presence of SDHB/D-related paraganglioma: initial experimental and ex vivo findings. Endocrinology 155 27–32. (10.1210/en.2013-1549) 24189137 PMC5398636

[bib52] Letouze E Martinelli C Loriot C Burnichon N Abermil N Ottolenghi C Janin M Menara M Nguyen AT Benit P, 2013 SDH mutations establish a hypermethylator phenotype in paraganglioma. Cancer Cell 23 739–752. (10.1016/j.ccr.2013.04.018) 23707781

[bib53] Leuthold P Schwab M Hofmann U Winter S Rausch S Pollak MN Hennenlotter J Bedke J Schaeffeler E & Haag M 2018 Simultaneous extraction of RNA and metabolites from single kidney tissue specimens for combined transcriptomic and metabolomic profiling. Journal of Proteome Research 17 3039–3049. (10.1021/acs.jproteome.8b00199) 30091608

[bib54] Li F He X Ye D Lin Y Yu H Yao C Huang L Zhang J Wang F Xu S, 2015 NADP(+)-IDH mutations promote Hypersuccinylation that impairs mitochondria respiration and induces apoptosis resistance. Molecular Cell 60 661–675. (10.1016/j.molcel.2015.10.017) 26585387

[bib55] Li M He Y Pang Y Zhang J Feng Y He Y Xu X Wei Y Zhong D Deng W, 2022 Somatic IDH1 hotspot variants in Chinese patients with pheochromocytomas and paragangliomas. Journal of Clinical Endocrinology and Metabolism 2022 dgac653. (10.1210/clinem/dgac653) 36355572

[bib56] Liu JY & Wellen KE 2020 Advances into understanding metabolites as signaling molecules in cancer progression. Current Opinion in Cell Biology 63 144–153. (10.1016/j.ceb.2020.01.013) 32097832 PMC7298879

[bib57] Liu Y Pang Y Caisova V Ding J Yu D Zhou Y Huynh TT Ghayee H Pacak K & Yang C 2020a Targeting NRF2-governed glutathione synthesis for SDHB-mutated pheochromocytoma and paraganglioma. Cancers (Basel) 12 280. (10.3390/cancers12020280) PMC7072390 31979226

[bib58] Liu Y Pang Y Zhu B Uher O Caisova V Huynh TT Taieb D Hadrava Vanova K Ghayee HK Neuzil J, 2020b Therapeutic targeting of SDHB-mutated pheochromocytoma/paraganglioma with pharmacologic ascorbic acid. Clinical Cancer Research 26 3868–3880. (10.1158/1078-0432.CCR-19-2335) 32152203 PMC7443979

[bib59] Lorendeau D Rinaldi G Boon R Spincemaille P Metzger K Jager C Christen S Dong X Kuenen S Voordeckers K, 2017 Dual loss of succinate dehydrogenase (SDH) and complex I activity is necessary to recapitulate the metabolic phenotype of SDH mutant tumors. Metabolic Engineering 43 187–197. (10.1016/j.ymben.2016.11.005) 27847310

[bib62] Lussey-Lepoutre C Hollinshead KE Ludwig C Menara M Morin A Castro-Vega LJ Parker SJ Janin M Martinelli C Ottolenghi C, 2015 Loss of succinate dehydrogenase activity results in dependency on pyruvate carboxylation for cellular anabolism. Nature Communications 6 8784. (10.1038/ncomms9784) PMC4632646 26522426

[bib61] Lussey-Lepoutre C Bellucci A Morin A Buffet A Amar L Janin M Ottolenghi C Zinzindohoue F Autret G Burnichon N, 2016 In vivo detection of succinate by magnetic resonance spectroscopy as a hallmark of SDHx mutations in paraganglioma. Clinical Cancer Research 22 1120–1129. (10.1158/1078-0432.CCR-15-1576) 26490314

[bib60] Lussey-Lepoutre C Bellucci A Burnichon N Amar L Buffet A Drossart T Fontaine S Clement O Benit P Rustin P, 2020 Succinate detection using in vivo (1)H-MR spectroscopy identifies germline and somatic SDHx mutations in paragangliomas. European Journal of Nuclear Medicine and Molecular Imaging 47 1510–1517. (10.1007/s00259-019-04633-9) 31834447

[bib63] Markley JL Bruschweiler R Edison AS Eghbalnia HR Powers R Raftery D & Wishart DS 2017 The future of NMR-based metabolomics. Current Opinion in Biotechnology 43 34–40. (10.1016/j.copbio.2016.08.001) 27580257 PMC5305426

[bib64] Martinelli S Amore F Mello T Mannelli M Maggi M & Rapizzi E 2022 Metformin treatment induces different response in pheochromocytoma/paraganglioma tumour cells and in primary fibroblasts. Cancers (Basel) 14 3471. (10.3390/cancers14143471) PMC9320533 35884532

[bib65] Marz J Kurlbaum M Roche-Lancaster O Deutschbein T Peitzsch M Prehn C Weismann D Robledo M Adamski J Fassnacht M, 2021 Plasma metabolome profiling for the diagnosis of catecholamine producing tumors. Frontiers in Endocrinology (Lausanne) 12 722656. (10.3389/fendo.2021.722656) PMC8453166 34557163

[bib66] Mete O Asa SL Gill AJ Kimura N De Krijger RR & Tischler A 2022 Overview of the 2022 WHO classification of paragangliomas and pheochromocytomas. Endocrine Pathology 33 90–114. (10.1007/s12022-022-09704-6) 35285002

[bib67] Moog S Salgues B Braik-Djellas Y Viel T Balvay D Autret G Robidel E Gimenez-Roqueplo AP Tavitian B Lussey-Lepoutre C, 2022 Preclinical evaluation of targeted therapies in Sdhb-mutated tumors. Endocrine-Related Cancer 29 375–388. (10.1530/ERC-22-0030) 35348472

[bib68] Morin A Goncalves J Moog S Castro-Vega LJ Job S Buffet A Fontenille MJ Woszczyk J Gimenez-Roqueplo AP Letouze E, 2020 TET-mediated hypermethylation primes SDH-deficient cells for HIF2alpha-driven mesenchymal transition. Cell Reports 30 4551–4566.e7. (10.1016/j.celrep.2020.03.022) 32234487

[bib69] Murakami M Sun N Greunke C Feuchtinger A Kircher S Deutschbein T Papathomas T Bechmann N William Wallace P Peitzsch M, 2021 Mass spectrometry imaging identifies metabolic patterns associated with malignant potential in pheochromocytoma and paraganglioma. European Journal of Endocrinology 185 179–191. (10.1530/EJE-20-1407) 33983135

[bib70] Osuna-Prieto FJ Martinez-Tellez B Ortiz-Alvarez L Di X Jurado-Fasoli L Xu H Ceperuelo-Mallafre V Nunez-Roa C Kohler I Segura-Carretero A, 2021 Elevated plasma succinate levels are linked to higher cardiovascular disease risk factors in young adults. Cardiovascular Diabetology 20 151. (10.1186/s12933-021-01333-3) PMC8314524 34315463

[bib71] Pamporaki C Prodanov T Meuter L Berends AMA Bechmann N Constantinescu G Beuschlein F Remde H Januszewicz A Kerstens MN, 2022 Determinants of disease-specific survival in patients with and without metastatic pheochromocytoma and paraganglioma. European Journal of Cancer 169 32–41. (10.1016/j.ejca.2022.03.032) 35500459

[bib72] Pan Y Mansfield KD Bertozzi CC Rudenko V Chan DA Giaccia AJ & Simon MC 2007 Multiple factors affecting cellular redox status and energy metabolism modulate hypoxia-inducible factor prolyl hydroxylase activity in vivo and in vitro. Molecular and Cellular Biology 27 912–925. (10.1128/MCB.01223-06) 17101781 PMC1800695

[bib74] Pang Y Lu Y Caisova V Liu Y Bullova P Huynh TT Zhou Y Yu D Frysak Z Hartmann I, 2018 Targeting NAD(+)/PARP DNA repair pathway as a novel therapeutic approach to SDHB-mutated cluster I pheochromocytoma and paraganglioma. Clinical Cancer Research 24 3423–3432. (10.1158/1078-0432.CCR-17-3406) 29636359 PMC7446242

[bib73] Pang Y Liu Y Pacak K & Yang C 2019 Pheochromocytomas and paragangliomas: from genetic diversity to targeted therapies. Cancers (Basel) 11 436. (10.3390/cancers11040436) PMC6521122 30925729

[bib75] Peitzsch M Novos T Kaden D Kurlbaum M Van Herwaarden AE Muller D Adaway J Grouzmann E Mcwhinney B Hoad K, 2021 Harmonization of LC-MS/MS measurements of plasma free normetanephrine, metanephrine, and 3-methoxytyramine. Clinical Chemistry 67 1098–1112. (10.1093/clinchem/hvab060) 33993248

[bib76] Perez K Jacene H Hornick JL Ma C Vaz N Brais LK Alexander H Baddoo W Astone K Esplin ED, 2022 SDHx mutations and temozolomide in malignant pheochromocytoma and paraganglioma. Endocrine-Related Cancer 29 533–544. (10.1530/ERC-21-0392) 35731023

[bib77] Pollard PJ Briere JJ Alam NA Barwell J Barclay E Wortham NC Hunt T Mitchell M Olpin S Moat SJ, 2005 Accumulation of Krebs cycle intermediates and over-expression of HIF1 alpha in tumours which result from germline FH and SDH mutations. Human Molecular Genetics 14 2231–2239. (10.1093/hmg/ddi227) 15987702

[bib78] Prejbisz A Lenders JW Eisenhofer G & Januszewicz A 2011 Cardiovascular manifestations of phaeochromocytoma. Journal of Hypertension 29 2049–2060. (10.1097/HJH.0b013e32834a4ce9) 21826022

[bib79] Pryma DA Chin BB Noto RB Dillon JS Perkins S Solnes L Kostakoglu L Serafini AN Pampaloni MH Jensen J, 2019 Efficacy and safety of high-specific-activity (131)I-MIBG therapy in patients with advanced pheochromocytoma or paraganglioma. Journal of Nuclear Medicine 60 623–630. (10.2967/jnumed.118.217463) 30291194 PMC6495236

[bib80] Rai SK Bril F Hatch HM Xu Y Shelton L Kalavalapalli S Click A Lee D Beecher C Kirby A, 2020 Targeting pheochromocytoma/paraganglioma with polyamine inhibitors. Metabolism: Clinical and Experimental 110 154297. (10.1016/j.metabol.2020.154297) PMC7482423 32562798

[bib81] Rao JU Engelke UF Rodenburg RJ Wevers RA Pacak K Eisenhofer G Qin N Kusters B Goudswaard AG Lenders JW, 2013 Genotype-specific abnormalities in mitochondrial function associate with distinct profiles of energy metabolism and catecholamine content in pheochromocytoma and paraganglioma. Clinical Cancer Research 19 3787–3795. (10.1158/1078-0432.CCR-12-3922) 23723300 PMC3715587

[bib82] Rao JU Engelke UF Sweep FC Pacak K Kusters B Goudswaard AG Hermus AR Mensenkamp AR Eisenhofer G Qin N, 2015 Genotype-specific differences in the tumor metabolite profile of pheochromocytoma and paraganglioma using untargeted and targeted metabolomics. Journal of Clinical Endocrinology and Metabolism 100 E214–E222. (10.1210/jc.2014-2138) 25459911 PMC5393507

[bib83] Remacha L Comino-Mendez I Richter S Contreras L Curras-Freixes M Pita G Leton R Galarreta A Torres-Perez R Honrado E, 2017 Targeted exome sequencing of Krebs cycle genes reveals candidate cancer-predisposing mutations in pheochromocytomas and paragangliomas. Clinical Cancer Research 23 6315–6324. (10.1158/1078-0432.CCR-16-2250) 28720665

[bib84] Ren X Diao X Zhuang J & Wu D 2022 Structural basis for the allosteric inhibition of hypoxia-inducible factor (HIF)-2 by belzutifan. Molecular Pharmacology 102 240–247. (10.1124/molpharm.122.000525) 36167425

[bib85] Richards S Aziz N Bale S Bick D Das S Gastier-Foster J Grody WW Hegde M Lyon E Spector E, 2015 Standards and guidelines for the interpretation of sequence variants: a joint consensus recommendation of the American College of Medical Genetics and Genomics and the Association for Molecular Pathology. Genetics in Medicine 17 405–424. (10.1038/gim.2015.30) 25741868 PMC4544753

[bib89] Richter S Peitzsch M Rapizzi E Lenders JW Qin N De Cubas AA Schiavi F Rao JU Beuschlein F Quinkler M, 2014 Krebs cycle metabolite profiling for identification and stratification of pheochromocytomas/paragangliomas due to succinate dehydrogenase deficiency. Journal of Clinical Endocrinology and Metabolism 99 3903–3911. (10.1210/jc.2014-2151) 25014000 PMC4184070

[bib86] Richter S D'antongiovanni V Martinelli S Bechmann N Riverso M Poitz DM Pacak K Eisenhofer G Mannelli M & Rapizzi E 2018 Primary fibroblast co-culture stimulates growth and metabolism in Sdhb-impaired mouse pheochromocytoma MTT cells. Cell and Tissue Research 374 473–485. (10.1007/s00441-018-2907-x) 30159755 PMC7350657

[bib87] Richter S Gieldon L Pang Y Peitzsch M Huynh T Leton R Viana B Ercolino T Mangelis A Rapizzi E, 2019 Metabolome-guided genomics to identify pathogenic variants in isocitrate dehydrogenase, fumarate hydratase, and succinate dehydrogenase genes in pheochromocytoma and paraganglioma. Genetics in Medicine 21 705–717. (10.1038/s41436-018-0106-5) 30050099 PMC6353556

[bib88] Richter S Klink B Nacke B De Cubas AA Mangelis A Rapizzi E Meinhardt M Skondra C Mannelli M Robledo M, 2016 Epigenetic mutation of the succinate dehydrogenase C promoter in a patient with two paragangliomas. Journal of Clinical Endocrinology and Metabolism 101 359–363. (10.1210/jc.2015-3856) 26652933

[bib90] Richter S Qiu B Ghering M Kunath C Constantinescu G Luths C Pamporaki C Bechmann N Meuter L Kwapiszewska A, 2022 Head/neck paragangliomas: focus on tumor location, mutational status and plasma methoxytyramine. Endocrine-Related Cancer 29 213–224. (10.1530/ERC-21-0359) 35171114 PMC8942340

[bib91] Riester A Weismann D Quinkler M Lichtenauer UD Sommerey S Halbritter R Penning R Spitzweg C Schopohl J Beuschlein F, 2015 Life-threatening events in patients with pheochromocytoma. European Journal of Endocrinology 173 757–764. (10.1530/EJE-15-0483) 26346138

[bib92] Sarkadi B Meszaros K Krencz I Canu L Krokker L Zakarias S Barna G Sebestyen A Papay J Hujber Z, 2020 Glutaminases as a novel target for SDHB-associated pheochromocytomas/paragangliomas. Cancers (Basel) 12 599. (10.3390/cancers12030599) PMC7139890 32150977

[bib93] Serena C Ceperuelo-Mallafre V Keiran N Queipo-Ortuno MI Bernal R Gomez-Huelgas R Urpi-Sarda M Sabater M Perez-Brocal V Andres-Lacueva C, 2018 Elevated circulating levels of succinate in human obesity are linked to specific gut microbiota. ISME Journal 12 1642–1657. (10.1038/s41396-018-0068-2) 29434314 PMC6018807

[bib94] Smestad J Erber L Chen Y & Maher LJ 2018 Chromatin succinylation correlates with active gene expression and is perturbed by defective TCA cycle metabolism. iScience 2 63–75. (10.1016/j.isci.2018.03.012) 29888767 PMC5993049

[bib95] Streiff RR & Bender JF 2001 Phase 1 study of N1-N11-diethylnorspermine (DENSPM) administered TID for 6 days in patients with advanced malignancies. Investigational New Drugs 19 29–39. (10.1023/a:1006448516938) 11291831

[bib96] Sulkowski PL Sundaram RK Oeck S Corso CD Liu Y Noorbakhsh S Niger M Boeke M Ueno D Kalathil AN 2018. Krebs-cycle-deficient hereditary cancer syndromes are defined by defects in homologous-recombination DNA repair. Nature Genetics 50 1086–1092. (10.1038/s41588-018-0170-4) 30013182 PMC6072579

[bib97] Tomlinson IP Alam NA Rowan AJ Barclay E Jaeger EE Kelsell D Leigh I Gorman P Lamlum H Rahman S, 2002 Germline mutations in FH predispose to dominantly inherited uterine fibroids, skin leiomyomata and papillary renal cell cancer. Nature Genetics 30 406–410. (10.1038/ng849) 11865300

[bib98] Ullrich M Richter S Liers J Drukewitz S Friedemann M Kotzerke J Ziegler CG Nolting S Kopka K & Pietzsch J 2023 Epigenetic drugs in somatostatin type 2 receptor radionuclide theranostics and radiation transcriptomics in mouse pheochromocytoma models. Theranostics 13 278–294. (10.7150/thno.77918) 36593963 PMC9800739

[bib99] van Faassen M Bischoff R Eijkelenkamp K De Jong WHA Van Der Ley CP & Kema IP 2020 In matrix derivatization combined with LC-MS/MS results in ultrasensitive quantification of plasma free metanephrines and catecholamines. Analytical Chemistry 92 9072–9078. (10.1021/acs.analchem.0c01263) 32484659 PMC7349590

[bib100] Vanharanta S Buchta M Mcwhinney SR Virta SK Peczkowska M Morrison CD Lehtonen R Januszewicz A Jarvinen H Juhola M, 2004 Early-onset renal cell carcinoma as a novel extraparaganglial component of SDHB-associated heritable paraganglioma. American Journal of Human Genetics 74 153–159. (10.1086/381054) 14685938 PMC1181902

[bib101] Varoquaux A Le Fur Y Imperiale A Reyre A Montava M Fakhry N Namer IJ Moulin G Pacak K Guye M, 2015 Magnetic resonance spectroscopy of paragangliomas: new insights into in vivo metabolomics. Endocrine-Related Cancer 22 M1–M8. (10.1530/ERC-15-0246) 26115958 PMC4609897

[bib102] Vyakaranam AR Crona J Norlen O Granberg D Garske-Roman U Sandstrom M Fross-Baron K Thiis-Evensen E Hellman P & Sundin A 2019 Favorable outcome in patients with pheochromocytoma and paraganglioma treated with 177Lu-DOTATATE. Cancers (Basel) 11 909. (10.3390/cancers11070909) PMC6678507 31261748

[bib103] Wallace PW Conrad C Bruckmann S Pang Y Caleiras E Murakami M Korpershoek E Zhuang Z Rapizzi E Kroiss M, 2020 Metabolomics, machine learning and immunohistochemistry to predict succinate dehydrogenase mutational status in phaeochromocytomas and paragangliomas. Journal of Pathology 251 378–387. (10.1002/path.5472) 32462735 PMC7548960

[bib104] William D Erdmann K Ottemoller J Mangelis A Conrad C Peitzsch M Schrock E Eisenhofer G Zacharis A Fussel S, 2021 Targeted quantification of carbon metabolites identifies metabolic progression markers and an undiagnosed case of SDH-deficient clear cell renal cell carcinoma in a German cohort. Metabolites 11 764. (10.3390/metabo11110764) PMC8624007 34822422

[bib105] Xian F Hendrickson CL & Marshall AG 2012 High resolution mass spectrometry. Analytical Chemistry 84 708–719. (10.1021/ac203191t) 22263633

[bib106] Xiao M Yang H Xu W Ma S Lin H Zhu H Liu L Liu Y Yang C Xu Y, 2012 Inhibition of alpha-KG-dependent histone and DNA demethylases by fumarate and succinate that are accumulated in mutations of FH and SDH tumor suppressors. Genes and Development 26 1326–1338. (10.1101/gad.191056.112) 22677546 PMC3387660

[bib107] Yang M & Pollard PJ 2013 Succinate: a new epigenetic hacker. Cancer Cell 23 709–711. (10.1016/j.ccr.2013.05.015) 23763995

[bib108] Yang M Ternette N Su H Dabiri R Kessler BM Adam J Teh BT & Pollard PJ 2014 The Succinated proteome of FH-mutant tumours. Metabolites 4 640–654. (10.3390/metabo4030640) 25105836 PMC4192685

[bib109] Yoo A Tang C Zucker M Fitzgerald K Dinatale RG Rappold PM Weiss K Freeman B Lee CH Schultz N, 2022 Genomic and metabolic hallmarks of SDH- and FH-deficient renal cell carcinomas. European Urology Focus 8 1278–1288. (10.1016/j.euf.2021.12.002) 35288096 PMC9464266

[bib110] Zheng L Mackenzie ED Karim SA Hedley A Blyth K Kalna G Watson DG Szlosarek P Frezza C & Gottlieb E 2013 Reversed argininosuccinate lyase activity in fumarate hydratase-deficient cancer cells. Cancer and Metabolism 1 12. (10.1186/2049-3002-1-12) PMC4108060 24280230

[bib111] Zhou G Myers R Li Y Chen Y Shen X Fenyk-Melody J Wu M Ventre J Doebber T Fujii N, 2001 Role of AMP-activated protein kinase in mechanism of metformin action. Journal of Clinical Investigation 108 1167–1174. (10.1172/JCI13505) 11602624 PMC209533

[bib112] Zuber SM Kantorovich V & Pacak K 2011 Hypertension in pheochromocytoma: characteristics and treatment. Endocrinology and Metabolism Clinics of North America 40 295–311, vii. (10.1016/j.ecl.2011.02.002) 21565668 PMC3094542

